# An Update on the Inflammatory Response after Endovascular Repair for Abdominal Aortic Aneurysm

**DOI:** 10.1155/2015/945035

**Published:** 2015-06-18

**Authors:** Eleni Arnaoutoglou, George Kouvelos, Andreas Koutsoumpelis, Nikolaos Patelis, Andreas Lazaris, Miltiadis Matsagkas

**Affiliations:** ^1^Department of Anesthesiology, School of Medicine, University of Ioannina, 45110 Ioannina, Greece; ^2^Department of Surgery, Vascular Surgery Unit, School of Medicine, University of Ioannina, 45110 Ioannina, Greece; ^3^First Department of Surgery, Vascular Surgery Division, School of Medicine, University of Athens, 11527 Athens, Greece; ^4^3rd Department of Surgery, Vascular Surgery Unit, Medical School, University of Athens, 11527 Athens, Greece

## Abstract

Postimplantation syndrome (PIS) is the clinical and biochemical expression of an inflammatory response following endovascular repair of an aortic aneurysm (EVAR). The goal of this review is to provide an update on the inflammatory response after endovascular repair of abdominal aortic aneurysm, discussing its causes and effects on the clinical outcome of the patient. PIS concerns nearly one-third of patients after EVAR. It is generally a benign condition, although in some patients it may negatively affect outcome. The different definitions and conclusions drawn from several studies reveal that PIS needs to be redefined with standardized diagnostic criteria. The type of the endograft's material seems to play a role in the inflammatory response. Future studies should focus on a better understanding of the underlying pathophysiology, predictors, and risk factors as well as determining whether effective preventive strategies are necessary.

## 1. Introduction

Abdominal aortic aneurysm (AAA) is a relatively common disease, with a reported incidence of up to 8% of men aged above 65 years. The indication for aneurysm treatment is the elimination of the risk of rupture and death. For asymptomatic patients elective repair of the aneurysm constitutes the most effective management to prevent rupture. However elective aortic surgery has been also associated with considerable risks and therefore elective AAA repair is not recommended until the risk of rupture exceeds the risks associated with the repair. For asymptomatic patients the risk of rupture exceeds the risk of repair when the AAA diameter is larger than 5–5.5 cm [1, 2].

Endovascular repair of AAA involves the placement of an expandable stent graft within the aorta to treat aortic disease without operating directly on the aorta through laparotomy. Initially, the promise of endovascular techniques to deliver a safer and at least equally effective AAA repair when compared to open repair seemed to be verified by a number of studies [3, 4]. The risk of a severe inflammatory response was reported to be lower than that of an open repair [5, 6]. However, recent reports have mentioned that endovascular procedures may initiate a systemic inflammatory response, known as postimplantation syndrome (PIS) [7–9]. This inflammatory process may be triggered by manipulations with sheaths and catheters within the aortic lumen and the intramural thrombus, leading to the release of inflammatory mediators such as tumor necrosis factor *α* (TNF-*α*), interleukin-6 (IL-6), and other cytokines [10, 11]. Endothelial dysfunction may also prompt this inflammatory reaction, reflecting a synergic role between the material of the graft and the endovascular surgical technique [7, 8]. Despite the fact that PIS is frequently well-tolerated by patients, its impact on the outcome of patients is still unknown, especially in patients at high risk, including the elderly with several comorbidities [12, 13]. According to the reporting standards for endovascular aneurysm repair (EVAR), PIS could be considered a moderate complication of the procedure [14]. This paper provides a review of the current knowledge on the inflammatory response after endovascular repair of AAA, discussing its relations with inflammatory biomarkers and its causes and effects on the clinical outcome of the patient.

## 2. AAA and Inflammation

Inflammation is considered a crucial component in the pathogenesis and development of AAA along with proteolytic processes, genetic coding, and alterations in wall tension. AAA has been described as a chronic proinflammatory condition [15]. Preceding reports concerning histological analysis of diseased aortas have all displayed the transmural infiltration of T and B cells, macrophages, dendritic cells neutrophils, and mast cells in the media and adventitia of aneurysmal aortas [16]. These cells generate cytokines in the affected abdominal wall. Although a vast asset of them has been researched, only a few are considered specific to this pathology as compared to normal or atherosclerotic aortas and characterize the inflammatory process in AAA. In particular, there is an overexpression of interleukin 6 and interleukin 8 and preeminence of their related responses, TNF-*α* and interferon gamma (INF-*γ*), which belong to the group of TH1 associated cytokines, IL1B and IL10 [17]. Furthermore, messenger RNA and protein analysis on AAA samples have shown a distinct presence of intense activation of inflammatory transcription factors nuclear factor *κ*B (NF-*κ*B) and activator-protein 1 (AP-1) in the smooth muscle cells [18]. This induces secretion of matrix metalloproteinases (MMPs) and other proteases leading to wall degradation and to repeated secretion of more cytokines thus perpetuating the inflammatory process.

In clinical application, there are several reports concerning measuring of plasma biomarkers in attempt to observe aneurysm progression such as C reactive protein (CRP) and IL. However, either results are conflicting or evidence is weak [19]. Karlsson et al. found no correlation of CRP and IL6 plasma levels with the aneurysm expansion rate, while De Haro et al. reported a statistically significant association of CRP levels with the growth rate of the aneurysm [20, 21]. Třeška et al. showed a significant correlation of IL-6 levels and AAA diameter and TNF-*α* with the symptoms of AAA [22]. Furthermore Jones et al. reported that for patients with small aneurysms a specific IL-6 genotype predicted future cardiovascular mortality without however finding any association between plasma IL-6 or IL-6 genotype and aneurysm growth [23]. AAA is a complex entity and despite the significant advances in surgical and endovascular approaches, no therapies have been established that can effectively retard the progressive inflammation and elastolysis. Concentrating on isolated components of the inflammatory process is unlikely to control aneurysm growth rate due to biological redundancy. Further research of the interactions and relations of the components of the inflammatory process as a whole assemblage and their clinical implication may have the potential to shift AAA detection and growth control in the future.

## 3. PIS: Incidence-Definition

Postimplantation syndrome is the clinical and biochemical expression of an inflammatory response following endovascular repair of an aortic aneurysm [24]. The reported incidence of PIS in the literature has been varying widely from 14 to 60% [25–28].

The underestimation of this syndrome, which is not systematically reported, and the relatively small number of patients involved in most studies may account for this variation. Additionally, the lack of a universally accepted definition should also be acknowledged. Velázquez et al. first described PIS in 1999 as the presence of fever and leukocytosis with a white blood cell (WBC) count >11,000/mL [24]. Thereafter Gorich et al. reported a 45% incidence of PIS by defining leukocytosis as WBC count >10,000/mL, while our group has recently reported a nearly 35% PIS rate with a WBC count cut-off value of 12,000/mL [9, 29]. Blum et al. reported a raised incidence of PIS (100%) in 154 consecutive EVAR patients, taking into account leukocytosis of WBC count >9800/mL and elevated CRP, but not the presence of fever [30].

Our group has proposed a definition of the syndrome according to the systemic inflammatory response (SIRS). This definition seems to be more reasonable because there is evidence supporting a systemic inflammatory response induced by the implantation of the endoprosthesis. PIS seems to constitute a SIRS state as it actually fulfills at least two of the SIRS criteria (fever and leukocytosis) [31]. We defined PIS as the presence of fever (>38°C) and leukocytosis (>12,000/*μ*L) according to the SIRS criteria. Based on current evidence we believe that this may signify the best way to associate variables related to the syndrome among different studies and to evaluate its clinical significance. However, hs-CRP values have also been strongly related to the presence of PIS and emerged as an important predictor of the 30-day outcome [9]. Voûte et al. have already defined PIS as a composite of a body temperature >38°C coinciding with CRP >10 mg/L [7]. In the light of our latest publication, it is likely that CRP may express more constantly and reliably the intensity of inflammatory response to the endograft deployment and might be included in the definition of the syndrome [9]. In any case a universally accepted definition is certainly necessary for use in the everyday clinical practice, as well as for reporting standards when comparing different studies.

PIS is mainly a clinical condition associated with the implantation of an endograft and is diagnosed by the presence of fever accompanied by elevated WBC count above normal without any evidence of an infection. In our practice no anti-inflammatory drugs (steroids or nonsteroids) are used routinely during the postoperative period. All patients presenting with fever during the postoperative period, whether or not fulfilling the PIS criteria, are undergoing a thorough work-up for possible infection. If any of these tests reveal evidence of an early pulmonary, urinary tract, or any other kind of infection, the patient is not considered to suffer from PIS. Patients are discharged in the absence of any complications, with a body temperature <37.5°C for at least 24 hours and a WBC <12.000/mL.

## 4. Causes

Multiple factors have been proposed as causes of the inflammatory response after EVAR. Some authors suggest that manipulation of the aneurysm may activate white blood cells and lead to release of various cytokines, while others speculate that injury to the endothelium may cause protein C activation and subsequent coagulopathy [11, 25, 32]. None of these theories have been proved. Furthermore, an experimental study showed that iodide-containing contrast agent that is used during EVAR for vessel visualization induced neutrophil granulocyte degranulation [33]. A PIS-induced effect due to contrast media has not been observed in either clinical study so far [7, 9, 34].

### 4.1. Thrombus

Aneurysm thrombus has also been proposed to incur a role on the inflammatory response. Norgren and Swartbol in the early years of EVAR proposed that this endovascular procedure may induce an inflammatory response mainly involving TNF-*α* release from cell activation arising from intra-aneurysmal device manipulation [27]. The theory was based on the finding that mural thrombus of an aortic aneurysm contains high amounts of IL-6 and that manipulations with endovascular instruments inside the mural thrombus might release IL-6 [10]. Gabriel et al. supported this hypothesis 10 years later, though neither of these studies performed any quantitative evaluation of thrombus manipulation during the procedure [26]. However, the amount of preexisting mural thrombus within the aneurysm sac was not found to have any association with the development of PIS in any recent study [7, 9, 34]. In a recent report our group found no differences either in total preoperative AAA volume or in the amount of newly formed thrombus between PIS and non-PIS group [9]. These results have also been confirmed by Voûte et al. in 136 patients, weakening the theory that the mural thrombus of the aneurysm was the source of the IL-6 amount in the circulation [7]. However, Kakisis et al. recently by reporting on 87 patients after EVAR found that the volume of new onset thrombus was associated with the development of the inflammatory response [34]. Future large studies should focus on the effect of sac thrombosis after AAA exclusion from the circulation on the inflammatory biomarkers and its relation with PIS development.

### 4.2. Role of Material

Differences between the type of the stent graft deployed and the development of PIS might indicate that different materials, and maybe configurations of the grafts, can interfere with an inflammatory response. Stent grafts are a collapsible hybrid product composed of either woven Dacron or ePTFE with stents providing for radial support. Gerasimidis et al. in a relatively underpowered study found for the first time in 2005 that fever was more common in a group of patients who received polyester endovascular grafts that in those that received PTFE graft [25]. IL-8 was higher in the first group, suggesting a stronger host reaction to the specific material. Voûte et al. in a later study showed that the implantation of stent grafts based on polyester was independently associated with a stronger inflammatory response [7]. Moulakakis et al., observing a milder inflammatory activation in patients with a PTFE endograft, have confirmed this finding in a later report [8]. Accordingly our group found that the use of polyester endograft independently predicted PIS and was correlated with an above 10 times higher risk for an inflammatory response [9]. Based on the results of the above 3 mentioned studies the type of endograft's material seems to incur a principle role in PIS development and may have a predictive role for a significant portion of EVAR patients.

Furthermore there are other differences between stent grafts, unrelated to graft material, which theoretically might also influence PIS occurrence. The vast majority of the stent grafts have an exoskeleton made of nitinol. Since the presentation of nitinol for medical application, it has been extensively used in coronary and peripheral arterial stents. No inflammatory response is reported in these applications, despite frequent treatment of multiple and lengthy lesions, requiring large quantities of the material. It is, therefore, doubtful that variances in the application of nitinol between stent grafts have any effect on PIS.

## 5. Role of C-Reactive Protein

One easily measurable factor for the determination of PIS is C-reactive protein (CRP). This is an acute phase protein, which, apart from reflecting the degree of systematic inflammation, might be considered as a surrogate of atherosclerotic burden that may also have a direct role in atherosclerotic plaque rupture and thrombosis [35, 36]. In a surgical setting Choi et al. reported that high-preoperative CRP represents a strong and independent predictor of perioperative major cardiovascular event in noncardiac surgery, while our group in a previous report suggested that hs-CRP may predict the occurrence of cardiovascular event during the first postoperative year after vascular surgery [37, 38]. CRP has been used as PIS indicator in many studies. CRP levels increase rapidly, usually 6 hours after an inflammatory trigger, reaches a peak in 1-2 days, and returns to baseline in 4 to 10 days [39]. Voûte et al. included CRP in PIS definition and described PIS as fever >38°C coinciding with an elevated serum CRP level above 10 mg/L [7].  hs-CRP values have been strongly related to the presence of PIS and also emerged as an important predictor of the 30-day outcome [9]. Although CRP seems to be able to serve as an indicator in the diagnosis of PIS, more studies are needed to confirm its real value.

## 6. Role of Cytokines

Cytokines seem to play an important role in the inflammatory response after EVAR. Cytokines constitute components of a complex signaling network. They can broadly get classified as growth factors, chemotactic factors (IL-4, IL-8), modulators of lymphocyte function (IL-2, IL-4), and modulators of the inflammatory response (IL-1b, TNF-*α*, and IL-6) [40, 41]. Swartbol et al. in an in vitro study of 10 mural thrombus specimens obtained from 10 different aortic aneurysms reported elevated levels of IL-6 from the aneurysmal thrombus, causing WBC stimulation and production of TNF-*α* [11]. This finding has been confirmed in a clinical study by Dawson et al. [42]. They found that circulating IL-6 was elevated within the aorta in patients with aneurysms and also correlated with the aneurysm surface area. In EVAR the endograft material is exposed to the bloodstream and its surface activates inflammatory mediators [7, 42]. Plasma levels of IL-6 after endoluminal graft placement in the abdominal aorta have been shown to increase in all patients on the first postoperative day [32, 43]. Gabriel et al. in a relatively small study (25 patients) evaluated the inflammatory reactions following endovascular abdominal and thoracic aortic aneurysm repair and showed that in all patients there was an elevation of IL-6 which culminated at 24 hours and triggered both an elevation of CRP and the development of fever 24 hours later [26]. Moulakakis et al. found significantly higher serum levels of IL-6, 8, 10 in all patients after EVAR [8]. In a pilot study from our group, we found a significant rise in IL-6 but not in IL-1 and TNF-*α* in PIS patients when compared to patients without PIS [43]. It is likely that IL-6 seems to express to some extent the inflammatory response after EVAR. However it is not a readily available assay in normal hospital laboratories and cannot be easily applied outside of research use.

## 7. Outcome

The relation of PIS with patient's outcome has not been adequately established. In most studies PIS is considered a benign condition, although it may lead to a more demanding postoperative care characterized by prolonged hospitalization [43]. Seldom, the inflammatory process has been reported to lead to the development of serious complications such as pulmonary dysfunction, cardiovascular events, renal insufficiency, and even multisystem organ failure [12, 44–46]. However, PIS has not been reported as an outcome measure in any of the large EVAR trials. Our group has reported a few years ago 5 EVAR and 1 TEVAR patients that needed readmission during the first 30 days after the procedure due to a systemic inflammatory response syndrome (SIRS) [12]. All these patients had PIS postoperatively and were discharged home after PIS had been attenuated. Several days later, they returned because of continuous fever and a generalized inflammatory response, including dyspnea, tachycardia, leg edema, weakness, anorexia, and even renal insufficiency and pleural infusions, which eventually led to readmission. All patients underwent several examinations and were assessed by experienced doctors within different specialties, but no other diagnosis founded by clinical evaluation and laboratory tests could be made, except that all patients were developing SIRS. Interestingly, none of the 113 patients who did not develop PIS after the endovascular procedure experienced such an inflammatory response that led to readmission. We concluded that, in some patients, the initial inflammatory response following EVAR is not always benign and therefore patients developing an excessive inflammation response may need close surveillance.

Nano et al. retrospectively reported on 118 patients after the deployment of specific endograft and did not find any association between the presence of PIS and the occurrence of long-term complications [47]. However the definition of the events was too “wide” including surgical and cardiovascular complications, while oddly only one of the patients sustained a major adverse cardiovascular event during a 4-year follow-up period. In the same study investigation of quality of life surveys showed that patients who had PIS after surgery felt significantly more limited in carrying out their daily physical activities and were more emotionally discouraged and depressed about their state of health than the group that did not have PIS. Moreover in a prospective study of 214 patients after EVAR this group found that patients with PIS were more likely to suffer from an adverse event during the 30 days after the procedure [9]. Adverse events occurred in 25.9% of the PIS group compared to 2.9% of the non-PIS group and included any major cardiovascular event, acute renal failure, readmission, and death by any cause. It is important that future studies assess whether PIS patients remain at a greater risk of suffering from an adverse event when compared to non-PIS patients even after the first month and might require closer surveillance after the procedure.

## 8. Do We Need to Intervene?

Considering the effect of PIS on patient's outcome the main question remains whether we should alter our approach and treat patients with PIS focusing on the diminution of the inflammatory response. Literature data are scarce and no therapeutic algorithm has ever been established. Gabriel et al. recommended aggressive use of anti-inflammatory drugs in the acute phase when patients present with extensive clinical signs of inflammation, while Morikage et al. prefer a more conservative approach [26, 32]. A group from Denmark published recently a randomized trial evaluating the effect of preoperative high-dose glucocorticoid on the inflammatory response and recovery after EVAR [48]. They included 150 patients that were randomized to receive preoperatively either 30 mg/kg of methylprednisolone (MP) or placebo. MP reduced systemic inflammatory response syndrome from 92% to 27% (*p* < 0.0001). Furthermore, maximal plasma interleukin 6 was also reduced from 186 pg/mL to 20 pg/mL (*p* < 0.001), while patients under MP had a shorter hospital stay (2 days versus 3 days, *p* < 0.001). However, no difference in 30-day surgical or medical morbidity was noted. Interestingly, Bischoff et al. in a recent survey of vascular surgery departments in Germany reported that 71% of the vascular centers treated PIS with nonsteroidal anti-inflammatory agents (NSAIDs) [28]. Still nonsteroidal anti-inflammatory drugs have been associated with increased cardiovascular morbidity and mortality and therefore cannot be given for long [49]. The anti-inflammatory properties of statins may be useful, though most EVAR patients are usually under statin treatment. It is reasonable that some patients with an intense inflammatory response might benefit from an anti-inflammatory therapeutic treatment, though this has not been proved in any study. In our practice we give oral anti-inflammatory drugs in all patients needing readmission due to vigorous inflammatory response and this has been proved to be an adequate treatment modality [12]. Future studies should focus on determining those patients that may benefit from any treatment by investigating the effect of routine or symptom-based anti-inflammatory therapy on qualitative and quantitative PIS characteristics and their relation with long-term outcome.

## 9. Conclusion

Postimplantation syndrome concerns nearly one-third of patients after EVAR. It is generally a benign condition, though in some patients it may negatively affect outcome. PIS needs to be redefined with standardized diagnostic criteria probably including other mediators of inflammation as CRP or IL-6. The type of the endograft's material seems to play a role in the inflammatory response. Future studies should focus on a better understanding of the underlying pathophysiology, predictors, and risk factors as well as determining whether preventive strategies are necessary.
